# Quantification of Stress- and Resistance-Related Metabolites in Barley Leaves (*Hordeum vulgare* L.) Infected with *Bipolaris sorokiniana* via UHPLC-MS/MS_MRM_

**DOI:** 10.1021/acs.jafc.6c01645

**Published:** 2026-06-16

**Authors:** Lisa Kurzweil, Timo D. Stark, Karina Hille, Felix Hoheneder, Jana Mrtva, Hans Hausladen, Miriam Lenk, Mohammed Saddik Motawie, Corina Vlot-Schuster, Klaus Pillen, Mette Sørensen, Birger Lindberg Møller, Ralph Hückelhoven, Corinna Dawid

**Affiliations:** † Professorship for Functional Phytometabolomics, TUM School of Life Sciences, 9184 Technical University of Munich, Lise-Meitner-Str. 34, Freising 85354, Germany; ‡ Chair of Food Chemistry and Molecular Sensory Science, TUM School of Life Sciences, 9184 Technical University of Munich, Lise-Meitner-Str. 34, Freising 85354, Germany; § Chair of Phytopathology, TUM School of Life Sciences, 9184 Technical University of Munich, Emil-Ramann-Str. 2, Freising 85354, Germany; ∥ Plant Technology Center, TUM School of Life Sciences, 9184 Technical University of Munich, Dürnast 9, Freising 85354, Germany; ⊥ Institute of Biochemical Plant Pathology, 26523 Helmholtz Zentrum München, Ingolstädter Landstraße 1, Neuherberg 85764, Germany; # Plant Biochemistry Laboratory, Department of Plant and Environmental Sciences, 4321 University of Copenhagen, Thorvaldsensvej 40, Frederiskberg C, Copenhagen 1871, Denmark; ∇ Chair of Crop Plant Genetics, Faculty of Life Sciences: Food, Nutrition and Health, University of Bayreuth, Fritz-Hornschuch-Straße 13, Kulmbach 95326, Germany; ○ Chair of Plant Breeding, Martin-Luther-University Halle-Wittenberg, Betty-Heimann-Str. 3, Halle (Saale) 06120, Germany; ◆ LEO Pharma, Industriparken 55, Ballerup, Copenhagen 5750, Denmark; ¶ Professorship for Chemosensory Food Systems, TUM School of Life Sciences, Technical University of Munich, Lise-Meitner-Str. 34, Freising 85354, Germany; ◘ Leibniz Institute for Food Systems Biology at the Technical University of Munich, Lise-Meitner-Str. 34, Freising 85354, Germany

**Keywords:** barley, *Bipolaris sorokiniana*, biotic stress, metabolites, LC-MS/MS, quantification

## Abstract

This study investigated the quantitative changes in 33 stress- or resistance-related metabolites induced by *Bipolaris sorokiniana* in barley leaves of quantitatively resistant and susceptible barley lines of the multiparental nested association mapping (NAM) population HEB-25. The analyses were based on ultrahigh-performance liquid chromatography tandem mass spectrometry (UHPLC-MS/MS). Twenty-nine infected and noninfected barley genotypes were analyzed at four different time points after inoculation. The method provided quantification of hordatines, phenolamides, hydroxycinnamic acids, flavone glucosides, hydroxynitrile glucosides, apocarotenoids, and indole derivatives. In leaves infected with *B. sorokiniana*, phenolamide levels were elevated compared to noninfected plants. A correlation between metabolite levels and the severity of infection showed that the more resistant barley lines contained higher amounts of hordatines and hordatine glucosides.

## Graphical abstract

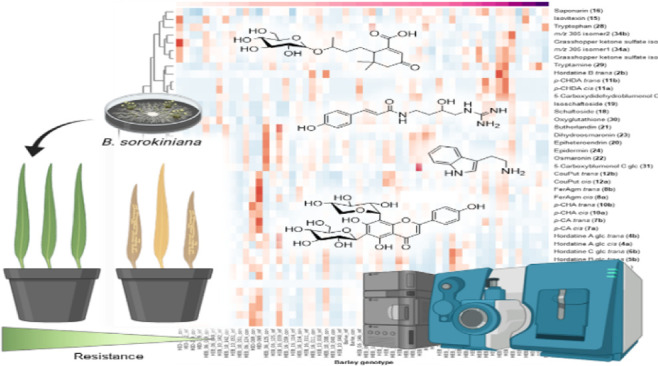

## Introduction

With a global cultivation area of nearly 70 million hectares, barley (*Hordeum vulgare* L.) ranks fourth among cereal crops according to production area.^1^ The harvested grain is mainly used as animal feed (65%), followed by malting (33%), and human consumption (2%).^2^ Barley cultivation is endangered by several biotic stresses, such as fungal infections. Among these, spot blotch caused by *Bipolaris sorokiniana* is one of the most common fungal diseases in barley^3,4^ with yield losses of 25–45%.^5^ Against the background of climate change and associated rising temperatures, fungal infections are increasing and causing pathogens to spread across the current Northern borders.^6,7^ The concomitant increased need for fertilizers and synthetic fungicides and the limitation of their use in the interests of consumers and the environment require crop species with improved resistance properties as well as the presence of alternative active constituents and naturally occurring defense molecules in plants.

Barley varieties with enhanced resistance to fungal infections have already been produced through plant breeding and several genes and quantitative trait loci (QTLs) causing pathogen resistance have been localized on different chromosomes.^5,8–18^

Plants respond to environmental stressors through the accumulation of defense-related specialized metabolites (bioactive natural products).^19,20^ Plant metabolites have diverse functions e.g., acting as signaling molecules, antioxidants, allelochemicals, direct insecticides, and antifungals. Depending on the plant cultivars, enhanced accumulation of plant metabolites may be associated with increased stress tolerance or pathogen resistance.^21,22^

In barley, barley-specific hordatines are known for their antifungal potential and contribution to resistance.^23,24^ Moreover, the biosynthetic precursors of hordatines, such as *p*-coumaroylagmatine (*p*-CA), which belong to the substance class of phenolamides or hydroxycinnamic acid amides, exhibit plant-protective properties.^25–27^ In barley leaves, hydroxynitrile glucosides inhibit fungal growth.^28^ Flavone glucosides and blumenol C glucosides are stress-induced metabolites in barley leaves in their response to abiotic stress.^29^ Tryptophan and tryptamine play an essential role in plant defense responses and stress tolerance to abiotic and biotic stress challenges.^30–33^ All of these substances as well as the previously identified carboxylated blumenol C glucosides and sulfated grasshopper ketone accumulate in barley leaves infected with *B. sorokiniana*, and some have shown antifungal activity against *B. sorokiniana* at 10 mmol/L.^34^

The transcriptome analysis by Basak et al. (2024) highlighted that the barley–*B. sorokiniana* interaction is governed by a complex, quantitative resistance involving extensive transcriptional reprogramming, such as upregulation of defense-related genes, including resistant gene analogs (RGAs), pathogenesis-related (PR) proteins, defensins, and disease resistance proteins.^35^

The aim of this study was to determine the quantitative content of those metabolites associated with biotic stress in barley leaves. The metabolite content was correlated with the quantitative resistance of different susceptible barley genotypes from the multiparental nested association mapping (NAM) population HEB-25.^36^ In a previous study, the HEB-25 lines showed quantitative resistance behavior toward *B. sorokiniana*.^34^ The significant differences in the severity of the infection among the 29 selected HEB-25 genotypes demonstrate the high genetic variability within the HEB-25 population and thus its suitability for the quantification of molecular resistance markers. This required development of an ultrahigh-performance liquid chromatography tandem mass spectrometry (UHPLC-MS/MS) method for the simultaneous quantitation of 33 marker metabolites in barley leaves (Figure 1).

**Figure 1:**
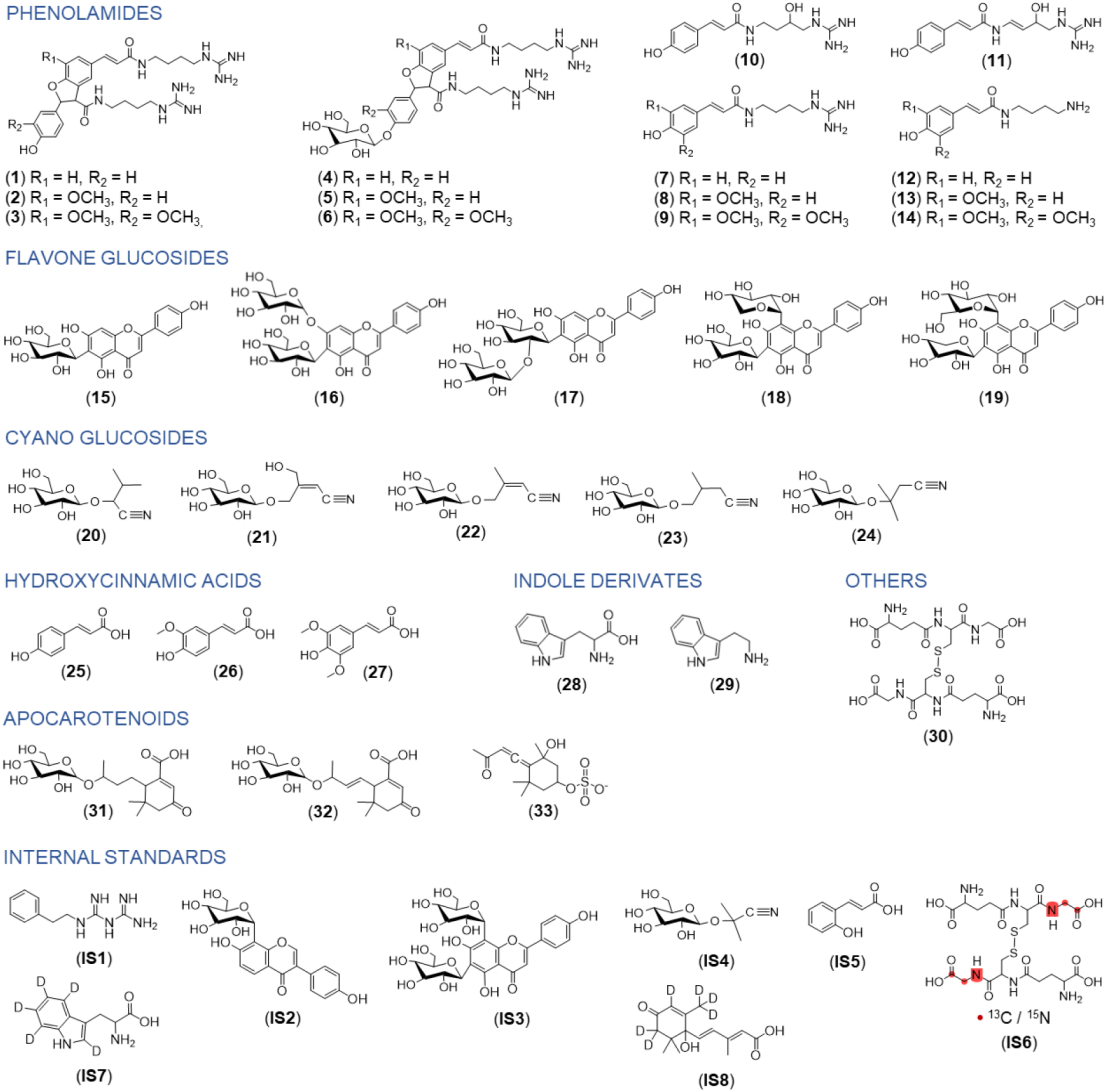
Chemical structures of marker metabolites (**1**–**33**) in barley leaves infected with *B. sorokiniana* and internal standards (**IS1**–**IS8**) used for the quantification of metabolites **1**–**33**. (**1**) hordatine A, (**2**) hordatine B, (**3**) hordatine C, (**4**) hordatine A glucoside, (**5**) hordatine B glucoside, (**6**) hordatine C glucoside, (**7**) *p*-coumaroylagmatine (*p*-CA), (**8**) feruloylagmatine (FerAgm), (**9**) sinapoylagmatine (SinAgm), (**10**) *p*-coumaroylhydroxylagmatine (*p*-CHA), (**11**) *p*-coumaroylhydroxydehydroagmatine (*p*-CHDA), (**12**) *p*-coumaroylputrescine, (**13**) feruloylputrescine (FerPut), (**14**) sinapoylputrescine (SinPut), (**15**) isovitexin, (**16**) saponarin, (**17**) meloside A, (**18**) schaftoside, (**19**) isoschaftoside, (**20**) epiheteroendrin, (**21**) sutherlandin, (**22**) osmaronin, (**23**) dihydroosmaronin, (**24**) epidermin, (**25**) *p*-coumaric acid, (**26**) ferulic acid, (**27**) sinapic acid, (**28**) L-tryptophan, (**29**) tryptamine, (**30**) oxyglutathione, (**31**) 5-carboxyblumenol C glucoside, (**32**) 5-carboxydidehydroblumenol C glucoside, (**33**) grasshopper ketone sulfate, (**IS1**) phenformin, (**IS2**) puerarin, (**IS3**) vicenin 2, (**IS4**) linamarin, (**IS5**) *o*-coumaric acid, (**IS6**) ^13^C_4_^15^N_2_-oxyglutathione, (**IS7**) tryptophan-*d*_5_, (**IS8**) abscisic acid-*d*_6_.

The method was fully validated for linearity, accuracy, precision, limit of detection (LOD), and limit of quantification (LOQ). This enabled analysis of 928 samples originating from 29 barley genotypes of the HEB-25 NAM population,^36^ infected with *B. sorokiniana* and noninfected controls at four different sampling times. Metabolite content/levels were correlated to the level of resistance or susceptibility of different barley lines as well as to the state of infection.

## Materials and Methods

### Chemicals

The reference substances isoschaftoside (**19**, ≥90%), *trans*-*p*-coumaric acid (**25**, ≥98%), *trans*-ferulic acid (**26**, 99%), *trans*-sinapic acid (**27**, ≥99%), L-tryptophan (**28**, ≥98%), tryptamine hydrochloride (**29**, 99%), l-glutathione oxidized (**30**, ≥ 98%), phenformin hydrochloride (**IS1**, ≥98%), and *trans*-*o*-coumaric acid (**IS5**, ≥98%) were obtained from Sigma-Aldrich (Steinheim, Germany). Isovitexin (**15**, ≥99%), saponarin (**16**, ≥98%), schaftoside (**18**, ≥90%), and puerarin (I**S2**, ≥98%) were purchased from Extrasynthese (Genay, France). Meloside A (**17**, >98%), ^13^C_4_^15^N_2_-oxyglutathione (**IS6**, 90%), and vicenin 2 (**IS3**, >99%) were obtained from MedChemExpress (Sollentuna, Sweden). Linamarin (**IS4**, >98%) was purchased from Cayman Chemicals (Ann Arbor, Michigan, USA), tryptophan-*d*_5_ (**IS7**, >95%), and abscisic acid-*d*_6_ (**IS8**, >95%) from Santa Cruz Biotechnology (Dallas, Texas, USA).

Hordatine A, B, and C (**1**–**3**), the corresponding hordatine glucosides (**4**–**6**), *p*-coumaroylhydroxyagmatine (*p*-CHA, **10**), and *p*-coumaroylhydroxydehydroagmatine (*p*-CHDA, **11**) were isolated from barley grains or leaves. Furthermore, *p*-coumaroylagmatine (*p*-CA, **7**), feruloylagmatine (FerAgm, **8**), sinapoylagmatine (SinAgm, **9**), *p*-coumaroylputrescine (CouPut, **12**), feruloylputrescine (FerPut, **13**), and sinapoylputrescine (SinPut, **14**) were synthesized, as previously noted.^34^ The hydroxynitrile glucosides **20**–**24** were synthesized by M. S. Motawia, as previously described^28^ and references were provided by M. Sørensen and B. L. Møller (University of Copenhagen, Denmark).

Acetonitrile, methanol, 2-propanol (Fisher Scientific, Schwerte, Germany), formic acid, and acetic acid (≥99%, HiPerSolv Chromanorm, VWR International, Darmstadt, Germany) were LC-MS grade. The water used for LC-MS was purified with an AQUA-Lab-B30-Integrity system (AQUA-Lab, Ransbach-Baumbach, Germany). The deuterated solvents D_2_O, DMSO-*d*_6_, and methanol-*d*_4_ were obtained from Sigma-Aldrich (Steinheim, Germany).

### Plant Material and Infection

A total of 29 barley lines from the nested association mapping (NAM) population HEB-25 ^36^ were selected, which differed genetically at the QTL QPt.4H-5, a candidate locus for net blotch (*Pyrenophora teres* *f. teres)* resistance.^37^ The respective wild barley parents (HID) and the recurrent parent Barke of the HEB-25 population were tested, too. Plants were cultivated under controlled conditions in a greenhouse cubicle with temperature control (18–20 °C heating temperature, 19–21 °C ventilation temperature, humidity 60–80%, and 16 h/day daylight exposure). Each genotype was grown in eight pots, of which four biological replicates were infected with *B. sorokiniana* by spray inoculation (10,000 spores/mL containing 1 mL Tween 80/L until runoff). Control plants were sprayed with distilled water (1 mL Tween80/L) until runoff. After inoculation, the plants were grown 3 days in a climate cabin (18 °C, 80% humidity, darkness) and sprayed several times with demineralized water to keep leaves wet and promote spore germination. For differentiation, the plants were returned to the greenhouse cubicle (16–18 °C heating temperature, 17–19 °C ventilation temperature, 60–80% relative humidity, daily irrigation). The visual symptoms on the leaves were characterized on a scale of 1 to 9 at 7, 10, 14, and 17 d after inoculation. A rating of 1 corresponds to a healthy plant without disease symptoms, and a rating of 9 corresponds to more than 80% infested leaf area. At all time points, leaf samples were taken and immediately frozen.

### Sample Preparation

Frozen barley leaves (50 mg) were weighed into bead beater tubes (2 mL, CKMix-2 mL, Bertin Technologies, Montigny-le-Bretonneux, France) filled with ceramic beads (zirconium oxide; mixture of 1.4 mm and 2.8 mm). After the addition of butylhydroxytoluene (25 μL, 1.0 mg/mL) an internal standard mixture (30 μL, IS) consisting of *o*-coumaric acid (**IS1**; 1.1 μmol/L), ^13^C_4_^15^N_2_-oxyglutathione (**IS2**; 1.1 μmol/L), puerarin (**IS3**; 2.0 μmol/L), vicenin 2 (**IS4**; 1.0 μmol/L), abscisic acid-*d*_6_ (**IS5**; 4.0 μmol/L), phenformin (**IS6**; 3.4 μmol/L), tryptophan-*d*_5_ (**IS7**; 1.7 μmol/L), linamarin (**IS8**; 1.2 μmol/L) and methanol/water (945 μL, 70/30, v/v), the mixture was homogenized in the Precellys homogenizer (Bertin Technologies, Montigny-le-Bretonneux, France; 6500 rpm, 3 × 30 s, 15 s pause) and equilibrated on a shaker (4 h, on ice, in the dark). After centrifugation, the supernatant was membrane filtered (0.45 μm; Minisart RC 15, Sartorius, Göttingen, Germany) and used for LC-MS/MS analysis.

### UHPLC-MS/MS Measurement

Quantification was performed using a Nexera X3 UHPLC (Shimadzu, Kyoto, Japan) coupled to a QTRAP 6500+ MS/MS system (Sciex, Darmstadt, Germany) in electrospray ionization (ESI) mode with positive and negative polarity switching. The UHPLC apparatus comprised of two LC pump systems (LC-40D X3), a DGU-405 degasser, a SIL-40C X3 autosampler, a CTO-40C column oven and an SCL-40 controller. Aliquots (1 μL) of the sample extracts were injected and separated on a Kinetex C_18_ column (2.1 mm × 100 mm; 1.7 μm; 100 Å; Phenomenex, Aschaffenburg, Germany) with an appropriate guard column. Chromatographic separation was performed at 40 °C with a flow rate of 0.4 mL/min. The mobile phase consisted of 0.1% formic acid in water (eluent A) and 0.1% formic acid in acetonitrile (eluent B). The composition of the mobile phase changed as follows: 1% B hold for 1 min, from 1 to 15% B in 7 min, from 15 to 20% B in 4 min, from 20 to 60% B in 2 min, from 60 to 100% B in 0.5 min, 100% B hold for 3 min, from 100 to 1% B in 0.5 min, and 1% B hold for 1 min. The autosampler temperature was set to 10 °C. The mass spectrometer was operated in scheduled multiple reaction monitoring (sMRM) mode (low mass) with the following device parameters: an ion spray voltage of −4500 V, curtain gas at 35 psi, a source temperature of 450 °C, nebulizer gas at 55 psi, heating gas at 65 psi, collision activated dissociation at −2 V, and a declustering potential of ±10 V. The MS parameters were optimized for each analyte and IS by the direct infusion of the pure reference substance solutions, and the most intensive mass transitions were selected as either quantifiers or qualifiers (Table S1). Data acquisition and device control were conducted using Analyst software (version 1.6.3; Sciex, Darmstadt, Germany), and data was undertaken using MultiQuant (version 3.0.2; Sciex, Darmstadt, Germany).

## Method Validation

### Linearity

Quantification was performed using internal calibration. The accurate concentration of each reference substance solution was verified by quantitative ^1^H NMR spectroscopy and aliquots of each solution were combined to form a calibration standard stock solution. The calibration stock solution was sequentially diluted 1 + 1 with acetonitrile/water (50/50, v/v) and an aliquot (30 μL) of the IS mixture was added to each dilution (970 μL). Twelve calibration points were recorded in the calibration range from 0.02–10 μmol/L (analyte **1**), from 0.01–5 μmol/L (analytes **2**–**6**), and from 0.05–100 μmol/L (analytes **7**–**10**, **12**–**16**, **18**–**31**,). Due to the limited availability of standard substances, the concentration of metabolite **17** was calculated with the calibration function of **16**, metabolite **32** was calculated as **31**, and metabolite **11** as **10**. The levels of metabolites **33** and **34** were only semiquantitatively assessed by comparing the area ratio of analyte and IS between the samples.

### Recovery Rate

As no analyte-free matrix was available, barley leaves were homogenized, spiked with three different concentration levels of the target analytes and further processed, as previously described. The endogenous concentrations present in the matrix before addition of the analytes were subtracted from the determined concentrations of each analyte. The recovery rate was calculated using the following formula:

$$Recovery\;rate=\frac{c_{\mathrm{measured}}-c_{\mathrm{endogen}}}{c_{\mathrm{spiked}}}\times 100\%$$

Three replicates were analyzed per concentration level.

### Precision

To determine the precision of this method, samples were prepared independently in triplicates on three different days (interday) or on the same day (intraday), and the coefficient of variation was calculated.

### Limit of Detection (LOD) and Limit of Quantification (LOQ)

A signal-to-noise ratio of 3 or 10 was chosen as the LOD or LOQ, respectively. The largest noise signal for each analyte represented via the mass transition of a barley matrix sample was integrated and the corresponding concentration was determined using the calibration function.^38^

### Data Analysis and Visualization

A visualization of quantitative data as heatmaps and correlation analysis was performed using MetaboAnalyst (version 6.0).^39^

### Quantitative ^1^H NMR (q^1^H NMR) Spectroscopy

The NMR measurements were carried out on an AVANCE III 400 MHz system (Bruker, Rheinstetten, Germany) equipped with an inverse BBI probe. To check the exact concentration of each analyte solution, the substances were dissolved in D_2_O (analytes **1**–**6**, **20**–**24**), methanol-*d*_4_ (analytes **7**–**10**, **12**–**14**, **18**, **25**–**26**, **28**–**30**) or DMSO-*d*_6_ (analytes **15**–**16**, **27**, **31**), and 600 μL of each solution was added to an NMR tube (5 mm × 178 mm; Bruker, Fällanden, Switzerland). The spectra were referenced to the solvent signal and expressed in parts per million (ppm). TopSpin 3.6 software (Bruker) was used for data processing. Quantitative ^1^H NMR spectroscopy was performed after calibration with the external calibration standards caffeine and tyrosine in D_2_O using the ERETIC II tool (Electronic REference To access In vivo Concentrations) and the PULCON method (PULse length based CONcentration determination).^40^

## Results and Discussion

### Quantification of Stress- and Resistance-Related Metabolites in Barley Leaves

A total of 29 differently resistant barley lines of the NAM population HEB-25 were grown and infected with *B. sorokiniana*.^36^ At 10, 14, and 17 d after inoculation, the severity of the spot blotch symptoms was phenotypically rated (disease scores are reported in literature^34^), and leaf samples were taken for LC-MS/MS analysis. In parallel, we examined parental HID and Barke lines.

In a previous study, we identified up- or downregulated metabolites in barley leaves after infection with *B. sorokiniana* and evaluated their potential antifungal activity against this fungus.^34^ To estimate whether the previously identified marker metabolites for an infection of barley with *B. sorokiniana* were present in barley leaves in sufficiently high concentrations to have an inhibitory effect against the pathogen and to investigate whether a quantitative relationship existed between metabolite concentrations and the resistance of the HEB-25 NAM population,^36^ we developed a quantitative UHPLC-MS/MS_MRM_ method. To compensate for analyte losses during sample preparation and matrix effects during ionization, eight structurally similar internal standards (IS) were used to quantify the 33 metabolites. The method was validated regarding linearity, accuracy, precision, LOD and LOQ (Tables S2–S3). The recovery rates for all analytes were in the range of 80–131%, and the precisions were between 0.2–26%. The LOD ranged from 0.001–0.366 μmol/L, and the LOQ ranged from 0.004–1.186 μmol/L.

The main metabolite in barley leaves was saponarin (**16**) at 0.055–4.46 μmol/g FW (Table S4). These values aligned with those in the literature.^41^ The parent line HID-219 showed the lowest concentrations of saponarin (**16**), but the highest concentrations of its isomer meloside A (**17**).

The content of hordatine aglycones (**1**–**3**) was higher at up to 2.72 μmol/g FW than the content of hordatine glucosides (**4**–**6**) at up to 0.085 μmol/g FW. The *cis*-isomers were higher than those of the *trans*-isomers in the barley leaves examined. Hordatine B (**2**) accounted for the largest proportion, followed by hordatine A (**1**) and C (**3**). To date, the literature has only provided quantitative information on hordatines (**1**–**3**) or hordatine glucosides (**4**–**6**) in beer and barley malt. The ratios of hordatines A (**1**), B (**2**) and C (**3**) in barley leaves determined in this study aligned with the values for beer and malt in the literature.^42,43^

Analogous to the hordatines (**1**–**6**), the content of the *cis*-isomers of the analyzed phenolamides (**7**–**14**) was higher than that of the *trans*-isomers. *Cis*- and *trans*-isomers of *p*-CHDA (**11**) have been described in literature and can be distinguished chromatographically.^44^ Reference substances were isolated from barley leaves and structurally characterized by 1D-/2-NMR experiment.^34^ The detection of multiple phenolamide isomers may arise from both biological and analytical sources. Biologically, isomerization can occur through enzymatic processes or spontaneous rearrangements, leading to compounds with potentially different biological activities. At the same time, nonenzymatic isomerization may take place during sample extraction, storage, or UHPLC-MS/MS analysis, particularly under conditions involving light exposure, pH variation, or elevated temperatures.

The biosynthetic precursors of hordatine A and B (**1**–**2**), *p*-CA (**7**), FerAgm (**8**), and *p*-CHA (**10**) dominated, whereas CouPut (**12**) and *p*-CHDA (**11**) were present in smaller amounts. SinAgm (**9**), FerPut (**13**), and SinPut (**14**) were undetectable in the samples. Ube et al. identified an increase in phenolamides (**7**–**8**, **10**–**13**) in *B. sorokiniana*-infected wheat leaves by using HPLC-UV and LC-MS/MS with external calibration.^45^ The method developed in this work represents the first LC-MS/MS-MRM method for the quantification of hordatines (**1**–**6**) and phenolamides (**7**–**14**) in barley leaves using authentic reference standards.

No comparable values could be found in the literature for 5-carboxyblumenol C 9-*O*-*ß*-d-glucoside (**31**) and the newly identified 5-carboxydidehydroblumenol C 9-*O*-*ß*-d-glucoside (**32**). The average values of 0.012 μmol/g FW were determined for **31** and 0.37 μmol/g FW for **32**, respectively.

The contents of the hydroxynitrile glucosides (**20**–**24**) ranged from 0.0092–4.09 μmol/g FW and aligned with the values described in the literature.^28,46–48^ The relative ratio of the hydroxynitrile glucosides, which has been found to be constant in many barley lines, was only partially reflected in the examined genotypes of the HEB-25 NAM population.^36,49^ The main proportion was epidermin (**24**) (52 ± 17%), followed by osmaronin (**22**) (20 ± 16%), epiheteroendrin (**20**) (15 ± 5%), sutherlandin (**21**) (9 ± 6%), and dihydroosmaronin (**23**), with 4 ± 7% of the total hydroxynitrile glucoside amount. The noninfected plants showed higher levels of hydroxynitrile glucosides than the infected barley leaves. This may reflect that some fungi are able to detoxify hydroxynitrile glucosides.^50^

The concentrations of tryptophan (**28**) ranged from 0.019–1.04 μmol/g FW, and tryptamine (**29**) ranged from 0.0040–0.94 μmol/g FW. Comparable values were found in *B. sorokiniana*-infected wheat leaves and in *B. oryzae*-inoculated rice leaves.^45,51^

Among the analyzed hydroxycinnamic acids (**25**–**27**), ferulic acid (**26**) was dominant in barley leaves, whereas *p*-coumaric acid (**25**) and sinapic acid (**27**) were only detected in trace amounts or were undetectable. This relative ratio was also established in many studies on barley grain.^52–55^ The low amount of free hydroxycinnamic acids in barley leaves could be due to this substance class being predominantly conjugated to cell wall components in plants.^56–59^

The natural concentrations of marker metabolites in barley leaves are significantly lower than the test solutions used in bioactivity tests for an inhibitory effect against *B. sorokiniana*.^34^ Since this study showed that some marker metabolites accumulate mainly in symptomatic areas, the local levels in symptomatic, pathogen-damaged leaf areas may be considerably higher. Whether additive or synergistic effects among different metabolites can occur must be clarified in follow-up studies.

### Resistance-Associated Marker Metabolites

We quantified the levels of marker metabolites in different resistant or susceptible barley lines of the HEB-25 NAM population and associated their levels with the severity of the symptoms of spot blotch caused by *B. sorokiniana*.^36^ The results of the earliest sampling time (7 d after inoculation) are shown in Figure 2, as the marker metabolites can be particularly useful for detecting stress-induced metabolome alterations in the early symptomatic stage of the disease.

**Figure 2:**
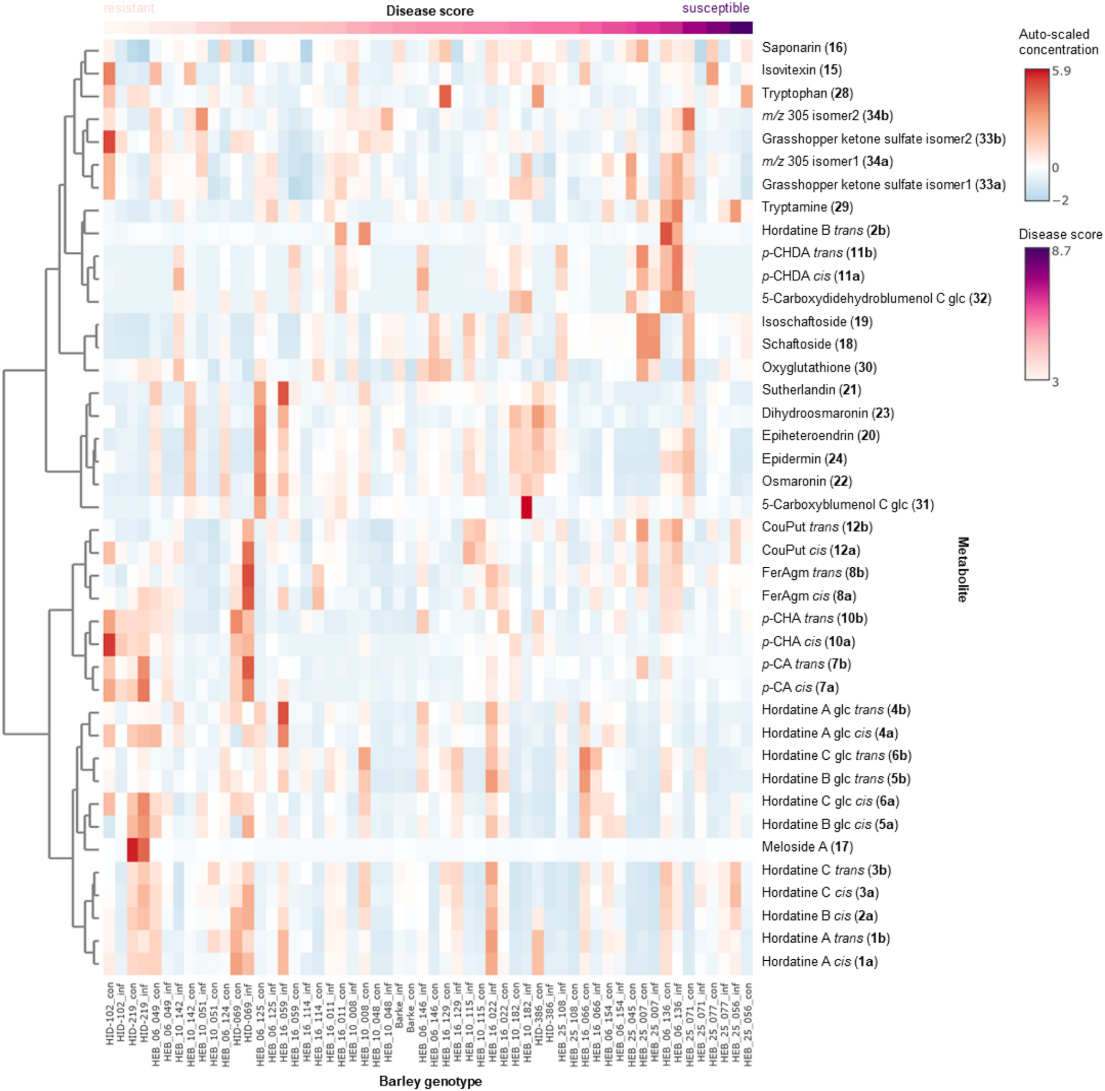
Hierarchical cluster analysis (HCA) of the quantitation of stress- and resistance-related marker metabolites in barley leaves of 29 selected genotypes of the HEB-25 NAM population.^36^ Sampling of infected (inf) and noninfected control (con) plants was carried out 7 days after inoculation with *B. sorokiniana*.

The results of the other sampling times can be found in the Supporting Information for this study (Figure S1).

In the more resistant barley lines, higher concentrations of hordatines (**1**–**3**), hordatine glucosides (**4**–**6**), and HCAA precursors (**7**–**9**) of hordatine biosynthesis were found (Figure 3).

**Figure 3:**
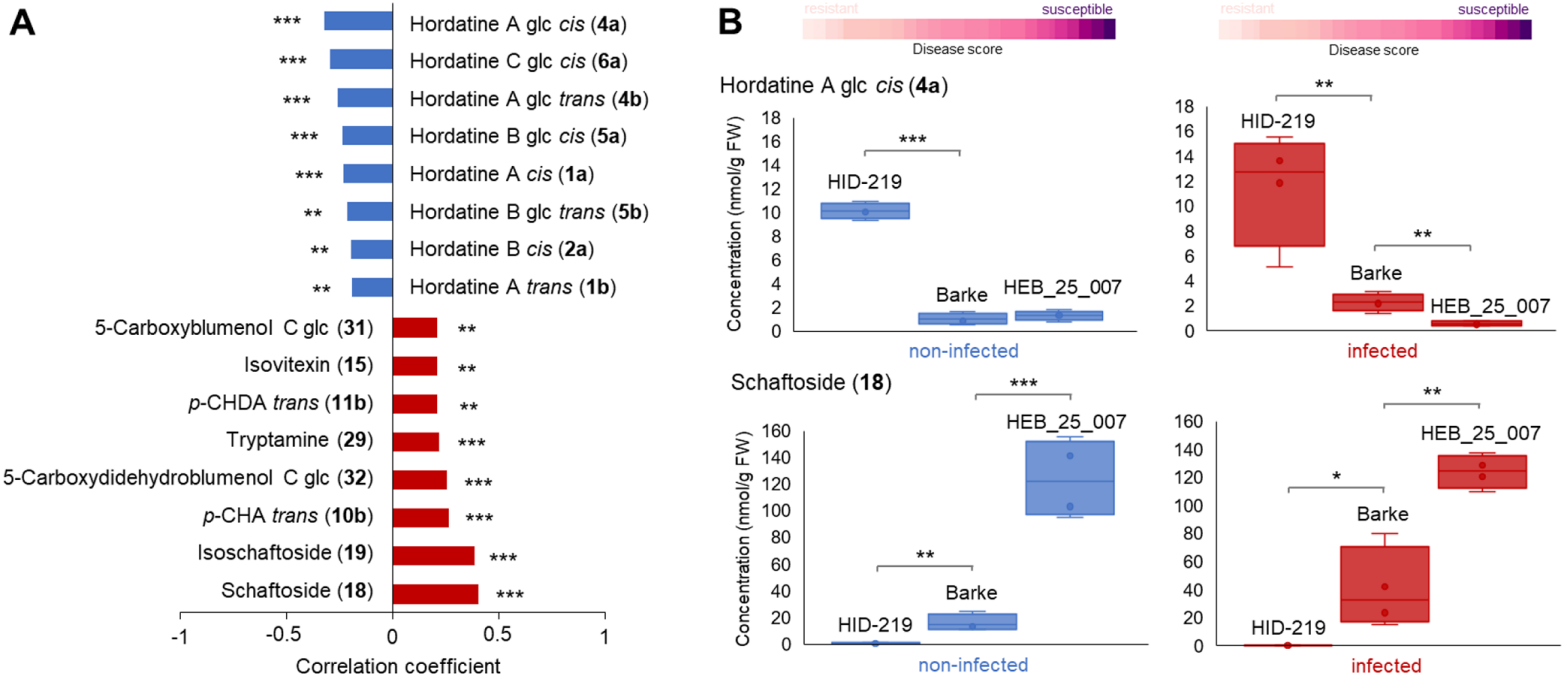
(A) Spearman rank correlation coefficients of the metabolites that most positively (red) and negatively (blue) correlate with the disease score of the *B. sorokiniana* infection of all tested genotypes. (B) Box plots of the metabolites that were most positively and negatively correlated with disease score of a resistant (left), medium-resistant (middle) and susceptible (right) barley genotypes. Sampling of infected (red) and noninfected control plants (blue) was carried out 7 days after inoculation with *B. sorokiniana*. *** *p* < 0.001; ** *p* < 0.01; * *p* < 0.1 (*n* = 4) (unpaired *t*-test).

In all barley lines, the content of hordatines (**1**–**3**) was lower in the infected plants than in the noninfected controls. An exception was the cultivar HEB_06_136, which was classified as relatively susceptible and showed high levels of hordatines (**1**–**3**) and the stress-related metabolites *p*-CHA (**10**), *p*-CHDA (**11**), tryptamine (**29**) and the previously identified apocarotenoids (**32**–**33**).

In contrast to previous reports,^25,60,61^ hordatines (**1**–**3**) did not clearly exhibit pathogen-induced upregulation in this study, and hordatine B (**2**) significantly decreased following infection. These results challenge the commonly proposed role of hordatines (**1**–**3**) as key contributors to disease resistance in barley. The reduction in hordatine B (**2**) may reflect complex metabolic regulation. It is possible that hordatine B (**2**) is converted into downstream metabolites or suppressed through feedback mechanisms within interconnected biosynthetic pathways. Additionally, shifts in metabolic flux under stress conditions may prioritize the production of other defense-related compounds. The lack of significant upregulation of hordatines (**1**–**3**) also suggests the involvement of broader regulatory networks. Plant defense responses are controlled by multiple signaling pathways, which may differentially modulate secondary metabolite biosynthesis depending on pathogen type and infection stage. Thus, hordatines (**1**–**3**) accumulation may not be universally induced under all conditions. Genotypic differences among barley varieties may further explain these observations. Distinct cultivars can exhibit variable metabolic responses. Comparative studies across other resistant and susceptible varieties could help to clarify this aspect. Finally, spatial heterogeneity in metabolite distribution may contribute to the observed patterns. Localized accumulation of hordatines (**1**–**3**) at infection sites could occur without detectable changes at the whole-tissue level, potentially obscuring their functional role. Overall, these findings suggest that the role of hordatines (**1**–**3**) in disease resistance is more complex and context-dependent than previously assumed, warranting further investigation into their regulation and function.

The more resistant lines had lower levels of the flavone glycosides saponarin (**16**), schaftoside (**18**), and isoschaftoside (**19**) at all time points compared to the more susceptible barley varieties. Likewise, the increased occurrence of tryptamine (**29**) was associated with susceptibility to *B. sorokiniana*.

To investigate a connection between the quantitative resistance of the examined genotypes and the metabolite content, the disease score of the *B. sorokiniana*-infection was correlated with the metabolite concentrations. Hordatines (**1**–**3**) and hordatine glucosides (**4**–**6**) were associated with quantitative resistance, whereas flavone glycosides (**15**, **18**–**19**), tryptamine (**29**), coumaroylagmatine derivatives (**10**–**11**), and blumenol C derivatives (**31**–**32**) were correlated with susceptibility (Figure 3). The later sampling time points showed similar results (Figure S2).

Hordatines (**1**–**3**) and phenolamides (**7**–**9**) quantified in this study originate from the phenylpropanoid pathway. Hordatines (**1**–**3**) are formed via oxidative coupling of hydroxycinnamoylagmatine precursors.^62,63^ Phenolamides have been implicated in plant resistance through multiple mechanisms, including direct antifungal activity, reinforcement of cell wall structures, and potential roles in defense signaling.^64–66^

While hordatines (**1**–**3**) and hordatine glucosides (**4**–**6**) showed negative correlations with disease severity, suggesting a potential role in resistance, these relationships remain associative. It is equally plausible that their accumulation reflects activation of broader defense pathways rather than a direct antifungal function. Previous studies have suggested antimicrobial or cell wall–reinforcing properties of such compounds, but their specific contribution to quantitative resistance against *B. sorokiniana* remains to be experimentally validated. Future research should therefore focus on functional approaches, such as the exogenous administration of purified metabolites, reverse genetics for the targeted modification of biosynthetic genes, or the use of near-isogenic lines with different metabolite accumulation to investigate causal relationships. The integration of metabolomics with transcriptomics or QTL mapping could also help to clarify whether these metabolites are drivers or markers of resistance.

### Infection-Associated Marker Metabolites

Even though the influence of the genotype on the metabolome was greater than that of inoculation with *B. sorokiniana*, we identified similarities in the up- or down-regulation of individual metabolites in response to the pathogen averaged over all time points (Figure 4).

**Figure 4:**
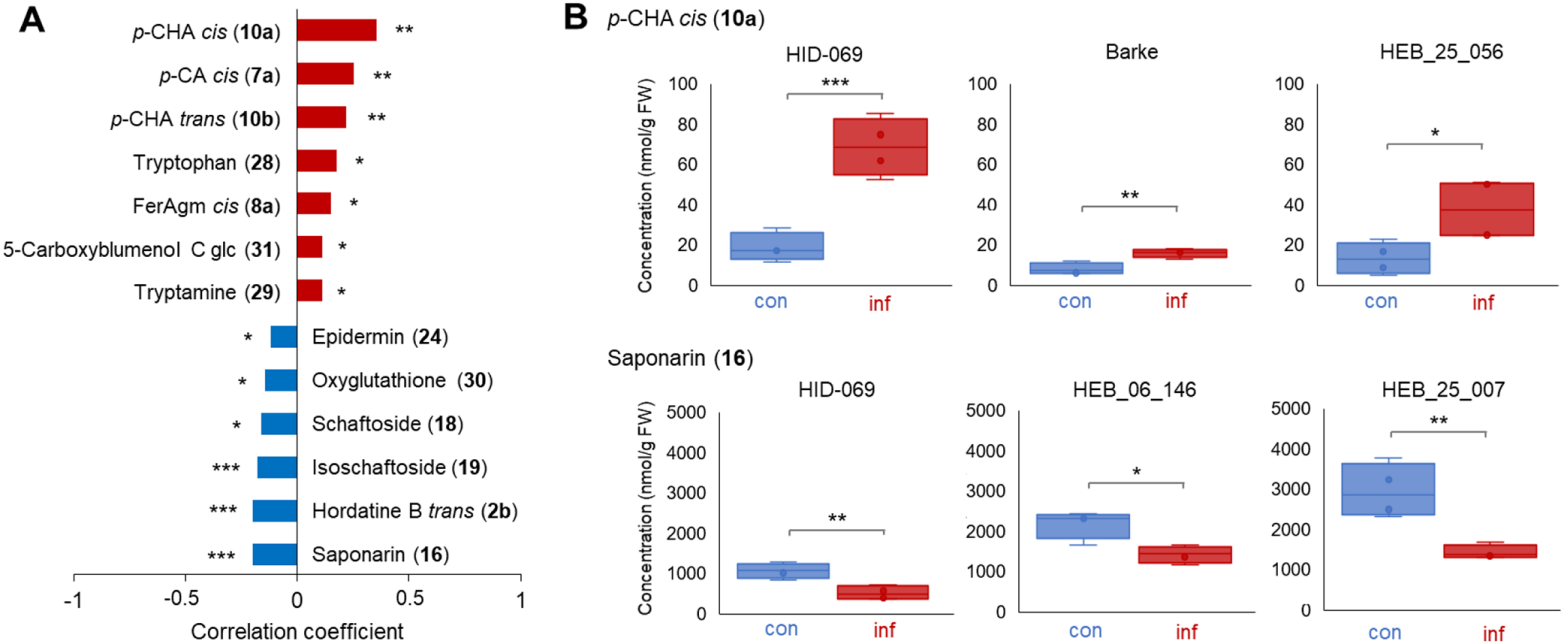
(A) Spearman rank correlation coefficients of the metabolites most positively (red) and negatively (blue) correlated with the *B. sorokiniana* infection (inoculated versus control). (B) Box plots of selected metabolites most positively and negatively correlated with the infection in a resistant (left), intermediately resistant (middle) and susceptible (right) barley line. Data of infected (inf) and noninfected control (con) plants shows average values of all four sampling time points. *** *p* < 0.001; ** *p* < 0.01; * *p* < 0.1; *n* = 116 (A); *n* = 16 (B) (unpaired *t*-test).

While coumaroylagmatines (**8**, **10**–**11**) and blumenol C derivatives (**31**–**32**) were positively correlated with the inoculation effect, flavone glycosides (**16**, **18**–**19**) and hordatines (**1**–**3**) were predominantly found in the healthy control plants (Figure 4A).

### Influence of the Time Point of Infection on Metabolite Levels

When considering the individual sampling times separately, we measured time-dependent up- or downregulation of marker metabolites (Figure S3). At the earliest sampling time (7 d after inoculation), tryptophan (**28**) was more abundant in the healthy control plants. In the course of the disease progression, tryptophan (**28**) levels increased in the infected leaves and decreased in the noninfected plants (Figure 5A).

**Figure 5:**
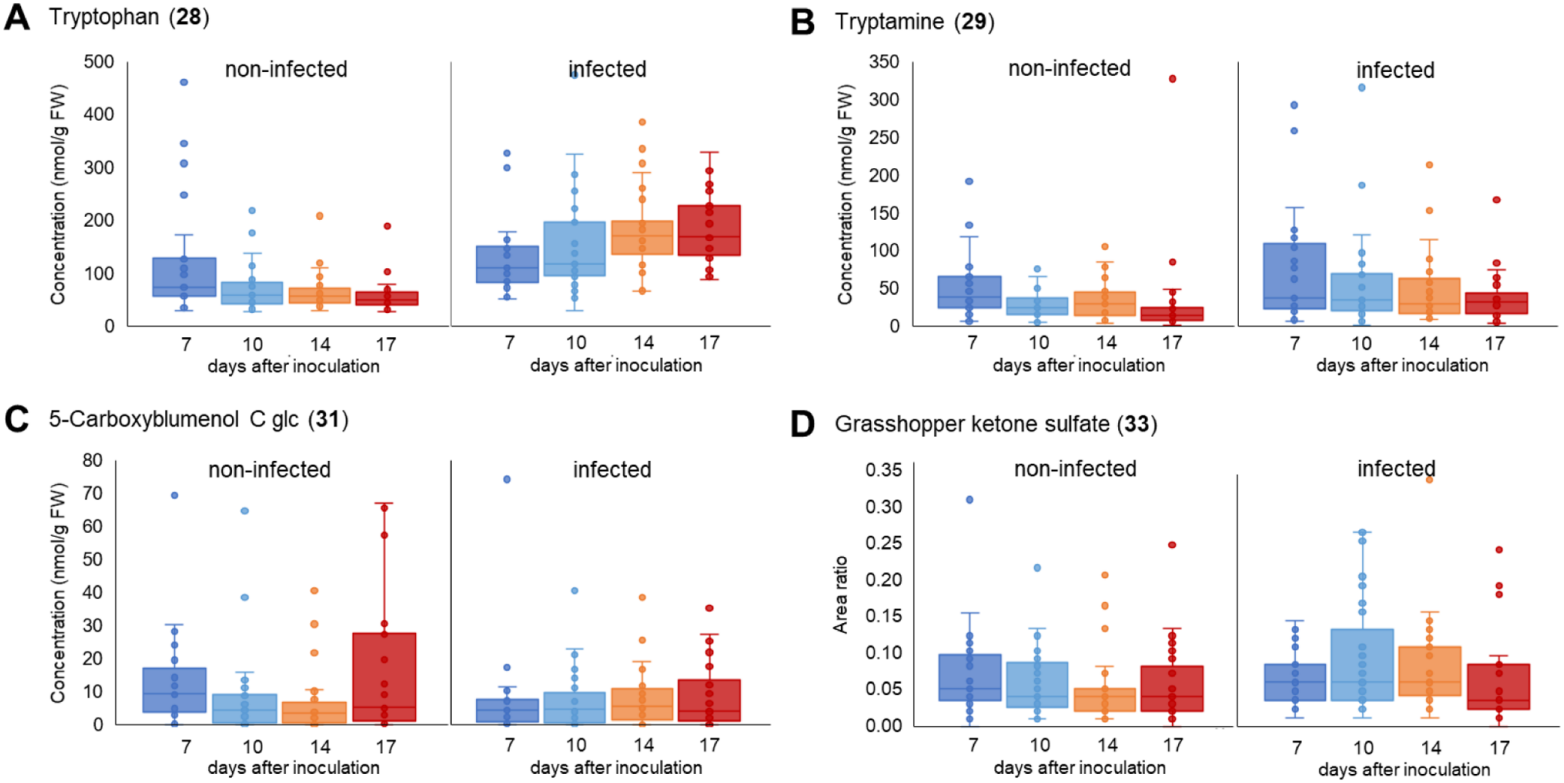
Box plots of selected metabolites (A–D) in all 29 examined barley cultivars of the HEB-25 NAM population^36^ at four different time points after inoculation with *B. sorokiniana* and noninfected controls.

In contrast, tryptamine (**29**) concentrations were more elevated in the infected plants than in the noninfected controls at all time points, but decreased in healthy and infected samples over time (Figure 5B). This could indicate stress-related upregulation of tryptophan biosynthesis and further conversion into tryptamine that additionally is plant age-dependently metabolized into other indole derivatives.

Similar behavior was observed for 5-carboxyblumenol C glucoside (**31**), and grasshopper ketone sulfate (**33**) for the first three time points (7, 10, 14 days post inoculation), when the levels decreased in the healthy plants, and increased in the infected plants (Figure 5C–D).

The quantitative resistance of the NAM population HEB-25 might be partially explained by the measured marker metabolites.^36^ However, the fluctuations between the biological replicates indicate that the observed symptoms of the *B. sorokiniana* infection were due to various influencing factors in addition to the metabolite concentrations. It should be also noted that positive correlations of metabolite concentrations with the degree of susceptibility or resistance cannot be readily interpreted as causative. Necrotic symptoms that develop in susceptible genotypes may associate with tissue damage that provokes secondary metabolite accumulation, perhaps even limiting more extreme pathogen spreading. e.g., both schaftoside (**18**) and tryptamine (**29**) were associated with disease susceptibility, but schaftoside (**18**) supported whereas tryptamine (**29**) inhibited *in vitro* growth of *B. sorokiniana*.^34^ Nevertheless, knowledge of the metabolic alterations in plants in response to biotic and abiotic stresses could contribute to the targeted control of diseases in future breeding programs. Biomarker molecules, as identified here, can be used in screenings of breeding material to detect diseases or resistance in the early, presymptomatic state of fungal infections.

In summary, we have successfully developed and validated a new LC-MS/MS_MRM_ method for quantification of a total of 33 metabolites in barley leaves, enabling the simultaneous measurement of a wide structural diversity of substances with high sensitivity. Quantitative values for hordatine A, B, and C (**1**–**3**), hordatine glycosides (**4**–**6**), flavone glycosides (**15**–**19**), oxyglutathione (**30**), phenolamides (**7**–**14**), the newly identified 5-carboxyblumenol-C-9-*O*-*ß*-d-glucoside (**31**), and 5-carboxydidehydroblumenol-C-9-*O*-*ß*-d-glucoside (**32**) were determined in barley leaves for the first time. The developed method allowed quantitative measurement of marker metabolites to be correlated with the quantitative resistance of selected HEB-25 genotypes and the quantitative determination of infection-induced upregulations of secondary metabolites. This method could be applied in the future to investigate the biotic stress resistance of various barley lines, other environmental and stress factors, or treatment effects. Further experiments with barley mutants, in which individual biosynthetic pathways for the formation of the identified resistance-related metabolites are silenced or overexpressed, could confirm that the metabolites associated with resistance in this study are responsible for their resistance. Comparative multiomics approaches will be essential to fully elucidate the dynamic host–pathogen interaction mechanisms.

## Supplementary Material



## Notes

MS parameters of the UHPLC-MS/MS-sMRM method; results of the validation of the UHPLC-MS/MS-sMRM method; calibration functions and the coefficient of determination (*R* ^2^); measured concentrations of the marker metabolites in the barley leaves of the HEB-25 NAM population; hierarchical cluster analyses (HCA) of the quantitation of marker metabolites in the barley leaves of 29 selected genotypes of the HEB-25 NAM population 10, 14, and 17 days after inoculation with *B. sorokiniana;* Spearman rank correlation coefficients of the metabolites that correlate with the disease score of the *B. sorokiniana* infection 10, 14, and 17 days after inoculation with *B. sorokiniana;* Spearman rank correlation coefficients of the metabolites correlated with infected and noninfected states 7, 10, 14, and 17 days after inoculation with *B. sorokiniana* (PDF)

## Abbreviations

*B. sorokiniana*: *Bipolaris sorokiniana*
*C*_*V*_: coefficient of variation
FA: formic acid
FerAgm: feruloylagmatine
FerPut: feruloylputrescine
FW: fresh weight
glc: glucoside
HCA: hierarchical cluster analysis
LC-MS: liquid chromatography coupled to mass spectrometry
LOD: limit of detection
LOQ: limit of quantification
*m*/*z*: mass-to-charge ratio
MRM: multiple reaction monitoring
*p*-CA: *p*-coumaroylagmatine
*p*-CHA: *p*-coumaroyl-3-hydroxyagmatine
*p*-CHDA: *p*-coumaroyl-3-hydroxydehydroagmatine
ppm: parts per million
qNMR: quantitative nuclear magnetic resonance
QTL: quantitative trait loci
SinAgm: sinapoylagmatine
SinPut: sinapoylputrescine
sMRM: scheduled multiple reaction monitoring
*t*_R_: retention time
