# Supplementary material for: Quantification of Stress- and Resistance-Related Metabolites in Barley Leaves (Hordeum vulgare L.) Infected with Bipolaris sorokiniana via UHPLC-MS/MSMRM
Source: J Agric Food Chem. 2026 Jun 16;74(25):20051–61. doi: 10.1021/acs.jafc.6c01645 (PMC13329990; doi:10.1021/acs.jafc.6c01645)
Supplement: Supplementary file 1 [file jf6c01645_si_001.pdf]

Supporting information

Quantification of stress- and resistance-related metabolites in barley leaves (*Hordeum vulgare* L.) infected with *Bipolaris sorokiniana* via UHPLC-MS/MS_{MRM}

Lisa Kurzweil,[1] Timo D. Stark,[2] Karina Hille,[2] Felix Hoheneder,[3] Jana Mrtva,[2] Hans Hausladen,[4] Miriam Lenk,[5] Mohammed Saddik Motawie,[6] Corina Vlot-Schuster,[5,7] Klaus Pillen,[8] Mette Sørensen,[6,9] Birger Lindberg Møller,[6] Ralph Hückelhoven,[3] Corinna Dawid,[1,2,10,11]*

[1] Professorship for Functional Phytometabolomics, TUM School of Life Sciences, Technical University of Munich, Lise-Meitner-Str. 34, 85354 Freising, Germany.

[2] Chair of Food Chemistry and Molecular Sensory Science, TUM School of Life Sciences, Technical University of Munich, Lise-Meitner-Str. 34, 85354 Freising, Germany.

[3] Chair of Phytopathology, TUM School of Life Sciences, Technical University of Munich, Emil-Ramann-Str. 2, 85354 Freising, Germany.

[4] Plant Technology Center, TUM School of Life Sciences, Technical University of Munich, Dürnast 9, 85354 Freising, Germany.

[5] Institute of Biochemical Plant Pathology, Helmholtz Zentrum München, Ingolstädter Landstraße 1, 85764 Neuherberg, Germany.

[6] Plant Biochemistry Laboratory, Department of Plant and Environmental Sciences, University of Copenhagen, Thorvaldsensvej 40, 1871 Frederiskberg C, Copenhagen, Denmark.

[7] Chair of Crop Plant Genetics, Faculty of Life Sciences: Food, Nutrition and Health, University of Bayreuth, Fritz-Hornschuch-Straße 13, 95326 Kulmbach, Germany.

[8] Chair of Plant Breeding, Martin-Luther-University Halle-Wittenberg, Betty-Heimann-Str. 3, 06120 Halle (Saale), Germany.

[9] LEO Pharma, Industriparken 55, 5750 Ballerup, Copenhagen, Denmark.

[10] Professorship for Chemosensory Food Systems, TUM School of Life Sciences, Technical University of Munich, Lise-Meitner-Str. 34, 85354 Freising, Germany.

[11] Leibniz Institute for Food Systems Biology at the Technical University of Munich, Lise-Meitner-Str. 34, 85354 Freising, Germany.

*** Corresponding author:**

Phone: +49 8161 712902
Fax: +49 8161 712949
E-mail: corinna.dawid@tum.de

Table S1. Optimized MS parameters of the UHPLC-MS/MS-sMRM method for the quantification of marker metabolites in barley leaves. The first mass transition of each substance served as a quantifier, the remaining mass transitions as qualifiers. The system was operated in polarity switch mode, in which ESI$^-$ and ESI$^+$ are measured simultaneously. IS internal standard, t_R retention time, EP entrance potential, CE collision energy, CXP collision exit potential.

No.	Substance name	mass transition Q1/Q3	t_R (min)	t_R window (s)	EP (V)	CE (V)	CXP (V)
**1**	hordatine A	276.1/131.0	7.8	120	11	25	20
**1**	hordatine A	276.1/265.1	7.8	120	11	27	24
**2**	hordatine B	291.1/131.1	7.7	100	51	25	16
**2**	hordatine B	291.1/277.9	7.7	100	51	41	24
**3**	hordatine C	306.2/131.1	8.1	90	1	25	16
**3**	hordatine C	306.2/157.0	8.1	90	1	25	18
**4**	hordatine A glucoside	357.2/276.2	7.8	60	61	23	24
**4**	hordatine A glucoside	357.2/290.9	7.8	60	61	35	32
**5**	hordatine B glucoside	372.2/291.2	7.7	60	26	25	24
**5**	hordatine B glucoside	372.2/295.0	7.7	60	26	37	24
**6**	hordatine C glucoside	387.2/306.1	8.0	60	61	25	16
**6**	hordatine C glucoside	387.2/350.9	8.0	60	61	37	40
**7**	*p*-CA	277.0/147.0	5.6	60	66	33	18
**7**	*p*-CA	277.0/91.0	5.6	60	66	63	10
**8**	feruloylagmatine	307.0/177.0	6.2	60	61	29	20
**8**	feruloylagmatine	307.0/144.9	6.2	60	61	43	16
**9**	sinapoylagmatine	337.0/207.0	6.5	60	76	29	24
**9**	sinapoylagmatine	337.0/175.0	6.5	60	76	39	20
**10**	*p*-CHA	293.0/147.0	4.8	90	111	31	26
**10**	*p*-CHA	293.0/275.1	4.8	90	111	21	26
**11**	*p*-CHDA	273.0/254.8	4.8	90	136	21	22
**11**	*p*-CHDA	273.0/146.9	4.8	90	136	31	16
**11**	*p*-CHDA	273.0/126.9	4.8	90	136	25	14
**12**	*p*-coumaroylputrescine	235.0/147.0	4.5	60	36	25	16
**12**	*p*-coumaroylputrescine	235.0/91.0	4.5	60	36	51	10
**13**	feruloylputrescine	365.1/177.0	9.5	60	36	25	20
**13**	feruloylputrescine	365.1/145.0	9.5	60	36	43	18
**14**	sinapoylputrescine	295.0/207.0	5.7	60	46	21	24
**14**	sinapoylputrescine	295.0/175.0	5.7	60	46	31	20
**15**	isovitexin	430.9/310.9	10.0	60	-45	-30	-39
**15**	isovitexin	430.9/341.0	10.0	60	-45	-32	-19
**16**	saponarin	593.1/310.9	8.6	60	-240	-50	-17
**16**	saponarin	593.1/296.9	8.6	60	-240	-60	-25
**17**	meloside A	593.0/293.0	9.4	60	-160	-46	-17
**17**	meloside A	593.0/412.8	9.4	60	-160	-34	-35
**18**	schaftoside	563.1/353.1	8.8	60	-130	-48	-19
**18**	schaftoside	563.1/382.9	8.8	60	-130	-50	-27

**19**	isoschaftoside	563.0/353.1	9.4	60	-65	-50	-23
**19**	isoschaftoside	563.0/382.9	9.4	60	-65	-50	-45
**20**	epiheteroendrin	283.9/284.0	5.0	90	126	9	22
**20**	epiheteroendrin	261.9/262.0	5.0	90	121	5	16
**21**	sutherlandin	297.9/297.9	1.5	90	41	9	18
**21**	sutherlandin	275.8/275.9	1.5	90	121	7	22
**22**	osmaronin	281.9/282.0	3.9	90	121	9	32
**22**	osmaronin	260.0/259.9	3.9	90	111	9	14
**23**	dihydroosmaronin	283.9/283.8	4.1	90	51	11	22
**23**	dihydroosmaronin	262.0/262.0	4.1	90	116	9	18
**24**	epidermin	283.9/284.1	3.2	90	141	9	18
**24**	epidermin	261.9/262.1	3.2	90	111	5	16
**24**	epidermin	261.9/203.9	3.2	90	111	19	24
**25**	*p*-coumaric acid	162.9/118.9	7.9	60	-75	-18	-17
**25**	*p*-coumaric acid	162.9/93.2	7.9	60	-75	-34	-15
**26**	ferulic acid	192.9/134.0	8.9	60	-40	-20	-15
**26**	ferulic acid	192.9/178.0	8.9	60	-40	-18	-19
**27**	sinapic acid	222.9/163.9	9.3	60	-10	-20	-17
**27**	sinapic acid	222.9/207.9	9.3	60	-10	-18	-9
**28**	tryptophan	205.0/187.9	4.6	90	1	13	22
**28**	tryptophan	205.0/145.8	4.6	90	1	25	18
**28**	tryptophan	205.0/117.9	4.6	90	1	33	14
**29**	tryptamine	160.9/143.9	5.1	90	86	15	16
**29**	tryptamine	160.9/116.9	5.1	90	86	27	14
**30**	oxyglutathione	611.0/305.9	1.6	90	-120	-34	-21
**30**	Oxyglutathione	611.0/271.8	1.6	90	-120	-36	-15
**31**	5-carboxyblumenol C glucoside	401.0/100.9	9.3	90	-100	-34	-9
**31**	5-carboxyblumenol C glucoside	401.0/221.0	9.3	90	-100	-30	-19
**32**	5-carboxydidehydroblumenol C glucoside	399.0/219.0	9.6	90	-60	-24	-19
**32**	5-carboxydidehydroblumenol C glucoside	399.0/174.9	9.6	90	-60	-32	-19
**33**	grashopper ketone sulfate	302.9/96.8	7.0	120	-25	-30	-11
**33**	grashopper ketone sulfate	302.9/79.9	7.0	120	-25	-102	-37
**34**	unknown ($C_{13}H_{21}SO_6$)	304.9/96.9	6.5	90	-65	-28	-13
**34**	unknown ($C_{13}H_{21}SO_6$)	304.9/79.9	6.5	90	-65	-106	-9
**IS1**	phenformin	206.0/60.0	5.4	90	26	21	14
**IS1**	phenformin	206.0/105.1	5.4	90	26	35	12
**IS2**	puerarin	415.0/267.1	7.4	60	-50	-48	-23
**IS2**	puerarin	415.0/294.8	7.4	60	-50	-32	-19
**IS3**	vicenin 2	593.0/353.0	8.0	60	-5	-48	-33
**IS3**	vicenin 2	593.0/383.1	8.0	60	-5	-50	-43
**IS4**	linamarin	269.9/270.3	2.7	90	111	13	10
**IS4**	linamarin	248.1/248.1	2.7	90	111	5	16
**IS5**	*o*-coumaric acid	162.9/118.9	9.9	60	-75	-18	-17
**IS5**	*o*-coumaric acid	162.9/93.2	9.9	60	-75	-34	-15
**IS6**	$^{13}C_4{}^{15}N_2$-oxyglutathione	617.0/308.8	1.6	90	-125	-34	-33
**IS6**	$^{13}C_4{}^{15}N_2$-oxyglutathione	617.0/274.9	1.6	90	-125	-38	-17

**IS7**	tryptophan-d_5	210.0/210.1	4.6	60	11	5	12
**IS7**	tryptophan-d_5	210.0/192.1	4.6	60	11	15	12
**IS8**	abscisic acid-d_6	268.9/159.1	13.2	60	-35	-16	-13
**IS8**	abscisic acid-d_6	268.9/225.0	13.2	60	-35	-20	-15

Table S2. Results of the validation of the UHPLC-MS/MS-sMRM method for the quantification of marker metabolites in barley leaves (n = 3). Recovery rate (mean values of three spiked concentrations), C_V coefficient of variation, LOD limit of detection, LOQ limit of quantification.

No.	Analyte	Internal standard (IS)	Recovery rate (%)	C_V (%) interday	C_V (%) intraday	LOD (µmol/L)	LOQ (µmol/L)
**1**	hordatine A	phenformin (**IS1**)	113	3.4	2.9	0.119	0.398
**2**	hordatine B	phenformin (**IS1**)	131	15.4	6.0	0.053	0.178
**3**	hordatine C	phenformin (**IS1**)	93	11.0	2.1	0.056	0.188
**4**	hordatine A glucoside	phenformin (**IS1**)	98	19.4	3.6	0.002	0.006
**5**	hordatine B glucoside	phenformin (**IS1**)	100	16.8	3.5	0.021	0.069
**6**	hordatine C glucoside	phenformin (**IS1**)	90	9.1	5.4	0.012	0.039
**7**	*p*-CA	phenformin (**IS1**)	88	0.2	5.0	0.056	0.185
**8**	feruloylagmatine	phenformin (**IS1**)	105	8.6	6.6	0.030	0.102
**9**	sinapoylagmatine	phenformin (**IS1**)	93	5.5	4.1	0.056	0.186
**10**	*p*-CHA	phenformin (**IS1**)	93	1.6	4.5	0.051	0.171
**12**	*p*-coumaroylputrescine	phenformin (**IS1**)	93	7.9	5.1	0.013	0.042
**13**	feruloylputrescine	phenformin (**IS1**)	117	11.3	4.3	0.137	0.456
**14**	sinapoylputrescine	phenformin (**IS1**)	80	17.0	5.0	0.108	0.360
**15**	isovitexin	puerarin (**IS2**)	110	13.4	5.1	0.075	0.250
**16**	saponarin	vicenin 2 (**IS3**)	102	8.9	6.8	0.011	0.038
**18**	schaftoside	puerarin (**IS2**)	123	11.3	9.2	0.078	0.101
**19**	isoschaftoside	puerarin (**IS2**)	100	9.3	6.7	0.053	0.061
**20**	epiheteroendrin	linamarin (**IS4**)	123	20.5	7.0	0.366	1.220
**21**	sutherlandin	linamarin (**IS4**)	112	12.2	3.8	0.361	1.205
**22**	osmaronin	linamarin (**IS4**)	115	9.7	1.4	0.311	1.037
**23**	dihydroosmaronin	linamarin (**IS4**)	108	26.2	5.9	0.178	0.594
**24**	epidermin	linamarin (**IS4**)	117	7.9	4.9	0.356	1.186
**25**	*p*-coumaric acid	*o*-coumaric acid (**IS5**)	112	12.6	4.2	0.014	0.045
**26**	ferulic acid	*o*-coumaric acid (**IS5**)	114	5.5	4.2	0.009	0.030
**27**	sinapic acid	*o*-coumaric acid (**IS5**)	97	14.7	6.3	0.049	0.163
**28**	tryptophan	tryptophan-d_5 (**IS7**)	99	8.8	2.0	0.001	0.004
**29**	tryptamine	tryptophan-d_5 (**IS7**)	112	14.4	6.7	0.003	0.011
**30**	oxyglutathione	$^{13}C_4{}^{15}N_2$-oxyglutathione (**IS6**)	99	15.3	2.9	0.005	0.018
**31**	5-carboxyblumenol C glucoside	abscisic acid-d_6 (**IS8**)	88	9.5	6.3	0.011	0.037

Table S3. Calibration functions and coefficient of determination (R^2) of the quantitation of marker metabolites in barley leaves using UHPLC-MS/MS.

No.	Analyte	Internal standard (IS)	Calibration function	R^2
**1**	hordatine A	phenformin (**IS1**)	y = 0.1401x	0.9926
**2**	hordatine B	phenformin (**IS1**)	y = 0.0559x	0.9913
**3**	hordatine C	phenformin (**IS1**)	y = 0.0327x	0.9919
**4**	hordatine A glucoside	phenformin (**IS1**)	$y = -0.0548x^2 + 0.6513x - 0.0141$	0.9971
**5**	hordatine B glucoside	phenformin (**IS1**)	$y = -0.0960x^2 + 1.007x - 0.0304$	0.9972
**6**	hordatine C glucoside	phenformin (**IS1**)	$y = -0.1191x^2 + 0.9009x - 0.0234$	0.9987
**7**	*p*-CA	phenformin (**IS1**)	y = 0.9399x	0.9961
**8**	feruloylagmatine	phenformin (**IS1**)	y = 0.0447x	0.9973
**10**	*p*-CHA	phenformin (**IS1**)	y = 0.2893x	0.9991
**12**	*p*-coumaroylputrescine	phenformin (**IS1**)	y = 0.1550x	0.9953
**13**	feruloylputrescine	phenformin (**IS1**)	y = 0.0019x	0.9969
**14**	sinapoylputrescine	phenformin (**IS1**)	y = 0.0270x	0.9975
**15**	isovitexin	puerarin (**IS2**)	y = 0.0992x	0.9956
**16**	saponarin	vicenin 2 (**IS3**)	y = 0.3737x	0.9977
**18**	schaftoside	puerarin (**IS2**)	y = 0.0501x	0.9979
**19**	isoschaftoside	puerarin (**IS2**)	y = 0.0638x	0.9985
**20**	epiheteroendrin	linamarin (**IS4**)	y = 0.5280x	0.9985
**21**	sutherlandin	linamarin (**IS4**)	y= 0.6656x	0.9952
**22**	osmaronin	linamarin (**IS4**)	y = 0.5017x	0.9977
**23**	dihydroosmaronin	linamarin (**IS4**)	y = 1.64365x	0.9924
**24**	epidermin	linamarin (**IS4**)	y = 0.5064x	0.9941
**25**	tryptophan	*o*-coumaric acid (**IS5**)	y = 0.3828x	0.9942
**26**	tryptamine	*o*-coumaric acid (**IS5**)	y = 0.1254x	0.9995
**27**	*p*-coumaric acid	*o*-coumaric acid (**IS5**)	y = 1.2875x	0.9997
**28**	ferulic acid	tryptophan-d_5 (**IS7**)	y = 0.4118x	0.9997
**29**	sinapic acid	tryptophan-d_5 (**IS7**)	y = 0.2917x	0.9994
**30**	oxyglutathione	$^{13}C_4{}^{15}N_2$-oxyglutathione (**IS6**)	y = 1.5783x	0.9994
**31**	5-carboxyblumenol C glucoside	abscisic acid-d_6 (**IS8**)	y = 0.0352x	0.9996

Table S4. Measured concentrations of marker metabolites in barley leaves of the HEB-25 NAM population (1).

No.	Analyte	Concentration (µmol/g FW)	
		Averge	Natural range
**1a**	hordatine A *cis*	0.044	< LOD – 0.95
**1b**	hordatine A *trans*	0.015	< LOD – 0.35
**2a**	hordatine B *cis*	0.21	< LOD – 2.72
**2b**	hordatine B *trans*	0.053	< LOD – 1.00
**3a**	hordatine C *cis*	0.093	< LOD – 0.84
**3b**	hordatine C *trans*	0.042	< LOD – 0.52
**4a**	hordatine A glucoside *cis*	0.0044	0.00040 – 0.073
**4b**	hordatine A glucoside *trans*	0.0034	0.00040 – 0.085
**5a**	hordatine B glucoside *cis*	0.011	< LOQ – 0.082
**5b**	hordatine B glucoside *trans*	0.0077	< LOQ – 0.075
**6a**	hordatine C glucoside *cis*	0.0041	< LOD – 0.056
**6b**	hordatine C glucoside *trans*	0.0022	< LOD – 0.035
**7a**	*p*-CA *cis*	0.012	< LOD – 0.14
**7b**	*p*-CA *trans*	0.0073	< LOD – 0.11
**8a**	feruloylagmatine *cis*	0.065	< LOD – 1.78
**8b**	feruloylagmatine *trans*	0.041	< LOD – 0.83
**10a**	*p*-CHA *cis*	0.013	< LOD – 0.17
**10b**	*p*-CHA *trans*	0.014	< LOD – 0.099
**11a**	*p*-CHDA *cis*	0.0030	< LOD – 0.062
**11b**	*p*-CHDA *trans*	0.0050	< LOD – 0.055
**12a**	*p*-coumaroylputrescine *cis*	0.0047	< LOD – 0.080
**12b**	*p*-coumaroylputrescine *trans*	0.00060	< LOD – 0.014
**15**	isovitexin	0.0080	< LOD – 0.085
**16**	saponarin	1.66	0.055 – 4.46
**17**	meloside A	0.10	< LOD – 4.12
**18**	schaftoside	0.041	< LOQ – 0.33
**19**	isoschaftoside	0.00078	< LOQ – 0.0075
**20**	epiheteroendrin	0.12	< LOD – 1.09
**21**	sutherlandin	0.080	< LOD – 1.47
**22**	osmaronin	0.13	< LOD – 1.52
**23**	dihydroosmaronin	0.0092	< LOD – 0.086
**24**	epidermin	0.080	< LOD – 4.09
**25**	*p*-coumaric acid	0.0021	< LOD – 0.025
**26**	ferulic acid	0.0091	< LOQ – 0.074
**27**	sinapic acid	0.00045	< LOD – 0.016
**28**	tryptophan	0.079	0.019 – 1.04
**29**	tryptamine	0.040	0.0040 – 0.94
**30**	oxyglutathione	0.059	0.0016 – 0.35
**31**	5-carboxyblumenol C glucoside	0.012	< LOD – 0.15

**32**	5-carboxydidehydroblumenol C glucoside	0.37	< LOQ – 4.85

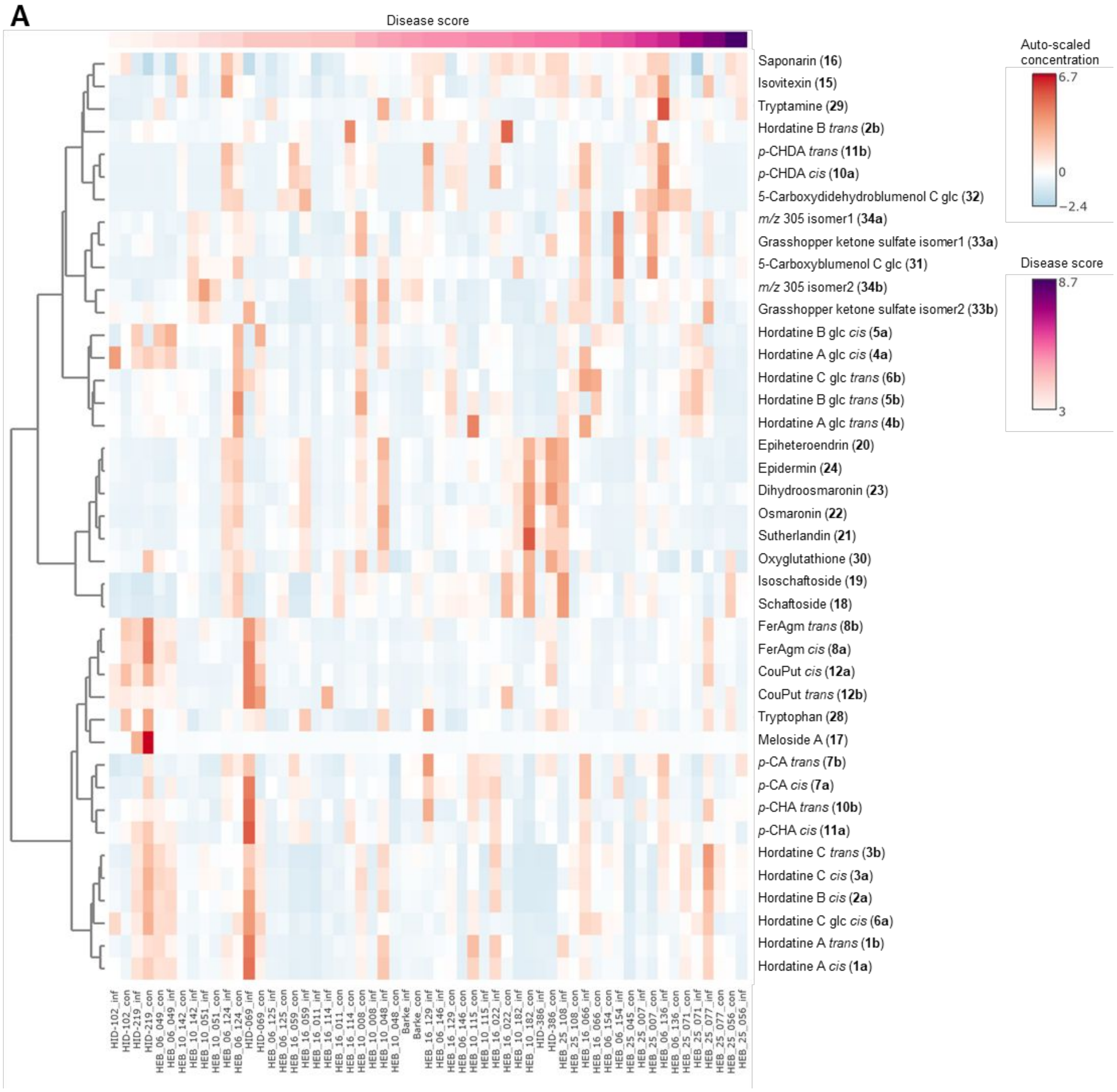
A
Disease score
Saponarin (16)
Isovitexin (15)
Tryptamine (29)
Hordatine B trans (2b)
p-CHDA trans (11b)
p-CHDA cis (10a)
5-Carboxydidehydroblumenol C glc (32)
m/z 305 isomer1 (34a)
Grasshopper ketone sulfate isomer1 (33a)
5-Carboxyblumenol C glc (31)
m/z 305 isomer2 (34b)
Grasshopper ketone sulfate isomer2 (33b)
Hordatine B glc cis (5a)
Hordatine A glc cis (4a)
Hordatine C glc trans (6b)
Hordatine B glc trans (5b)
Hordatine A glc trans (4b)
Epiheteroendrin (20)
Epidermin (24)
Dihydroosmaronin (23)
Osmaronin (22)
Sutherlandin (21)
Oxyglutathione (30)
Isoschaftoside (19)
Schaftoside (18)
FerAgm trans (8b)
FerAgm cis (8a)
CouPut cis (12a)
CouPut trans (12b)
Tryptophan (28)
Meloside A (17)
p-CA trans (7b)
p-CA cis (7a)
p-CHA trans (10b)
p-CHA cis (11a)
Hordatine C trans (3b)
Hordatine C cis (3a)
Hordatine B cis (2a)
Hordatine C glc cis (6a)
Hordatine A trans (1b)
Hordatine A cis (1a)
Auto-scaled concentration
6.7
0
−2.4
Disease score
8.7
3
HID-102_inf
HID-102_con
HID-219_inf
HID-219_con
HEB_06_049_con
HEB_06_049_inf
HEB_10_142_con
HEB_10_142_inf
HEB_10_051_inf
HEB_10_051_con
HEB_06_124_inf
HEB_06_124_con
HID-069_inf
HID-069_con
HEB_06_125_inf
HEB_06_125_con
HEB_16_059_con
HEB_16_059_inf
HEB_16_011_inf
HEB_16_114_inf
HEB_16_011_con
HEB_16_114_con
HEB_10_008_con
HEB_10_008_inf
HEB_10_048_inf
HEB_10_048_con
Barke_inf
Barke_con
HEB_16_129_inf
HEB_06_146_inf
HEB_16_129_con
HEB_06_146_con
HEB_10_115_con
HEB_10_115_inf
HEB_16_022_inf
HEB_16_022_con
HEB_10_182_inf
HEB_10_182_con
HID-386_inf
HID-386_con
HEB_25_108_inf
HEB_25_108_con
HEB_16_066_inf
HEB_16_066_con
HEB_06_154_con
HEB_06_154_inf
HEB_25_045_con
HEB_25_007_inf
HEB_25_007_con
HEB_06_136_inf
HEB_06_136_con
HEB_25_071_con
HEB_25_071_inf
HEB_25_077_inf
HEB_25_077_con
HEB_25_056_con
HEB_25_056_inf

B

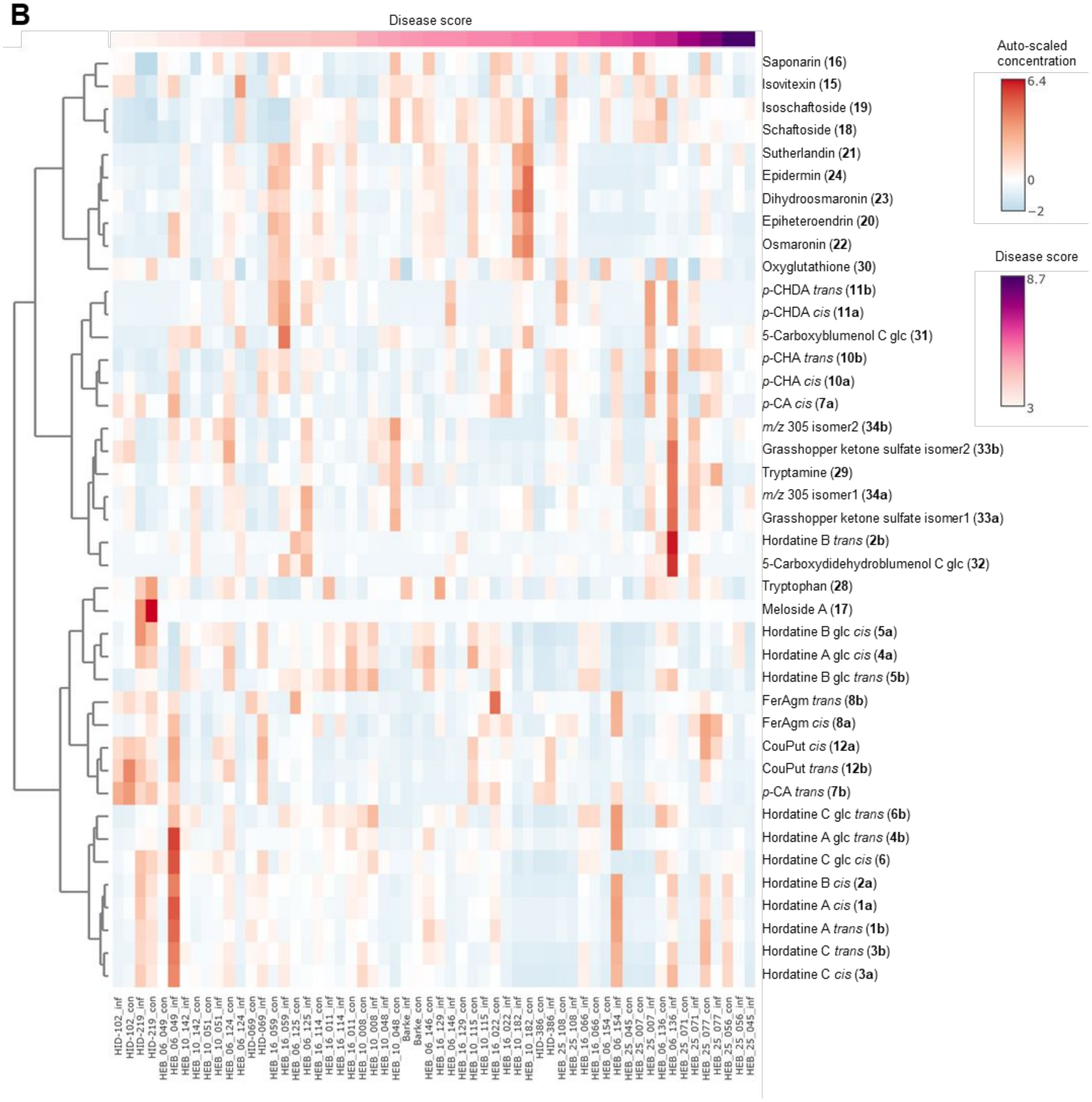
Disease score
Saponarin (16)
Isovitexin (15)
Isoschaftoside (19)
Schaftoside (18)
Sutherlandin (21)
Epidermin (24)
Dihydroosmaronin (23)
Epiheteroendrin (20)
Osmaronin (22)
Oxyglutathione (30)
p-CHDA trans (11b)
p-CHDA cis (11a)
5-Carboxyblumenol C glc (31)
p-CHA trans (10b)
p-CHA cis (10a)
p-CA cis (7a)
m/z 305 isomer2 (34b)
Grasshopper ketone sulfate isomer2 (33b)
Tryptamine (29)
m/z 305 isomer1 (34a)
Grasshopper ketone sulfate isomer1 (33a)
Hordatine B trans (2b)
5-Carboxydidehydroblumenol C glc (32)
Tryptophan (28)
Meloside A (17)
Hordatine B glc cis (5a)
Hordatine A glc cis (4a)
Hordatine B glc trans (5b)
FerAgm trans (8b)
FerAgm cis (8a)
CouPut cis (12a)
CouPut trans (12b)
p-CA trans (7b)
Hordatine C glc trans (6b)
Hordatine A glc trans (4b)
Hordatine C glc cis (6)
Hordatine B cis (2a)
Hordatine A cis (1a)
Hordatine A trans (1b)
Hordatine C trans (3b)
Hordatine C cis (3a)
Auto-scaled concentration
6.4
0
−2
Disease score
8.7
3
HID-102_inf
HID-102_con
HID-219_inf
HID-219_con
HEB_06_049_con
HEB_06_049_inf
HEB_10_142_inf
HEB_10_142_con
HEB_10_051_con
HEB_10_051_inf
HEB_06_124_con
HEB_06_124_inf
HID-069_con
HID-069_inf
HEB_16_059_con
HEB_16_059_inf
HEB_06_125_con
HEB_06_125_inf
HEB_16_114_con
HEB_16_011_inf
HEB_16_114_inf
HEB_16_011_con
HEB_10_008_con
HEB_10_008_inf
HEB_10_048_inf
HEB_10_048_con
Barke_inf
Barke_con
HEB_06_146_con
HEB_16_129_inf
HEB_06_146_inf
HEB_16_129_con
HEB_10_115_con
HEB_10_115_inf
HEB_16_022_con
HEB_16_022_inf
HEB_10_182_inf
HEB_10_182_con
HID-386_con
HID-386_inf
HEB_25_108_con
HEB_25_108_inf
HEB_16_066_inf
HEB_16_066_con
HEB_06_154_con
HEB_06_154_inf
HEB_25_045_con
HEB_25_007_con
HEB_25_007_inf
HEB_06_136_con
HEB_06_136_inf
HEB_25_071_con
HEB_25_071_inf
HEB_25_077_con
HEB_25_077_inf
HEB_25_056_con
HEB_25_056_inf
HEB_25_045_inf

C

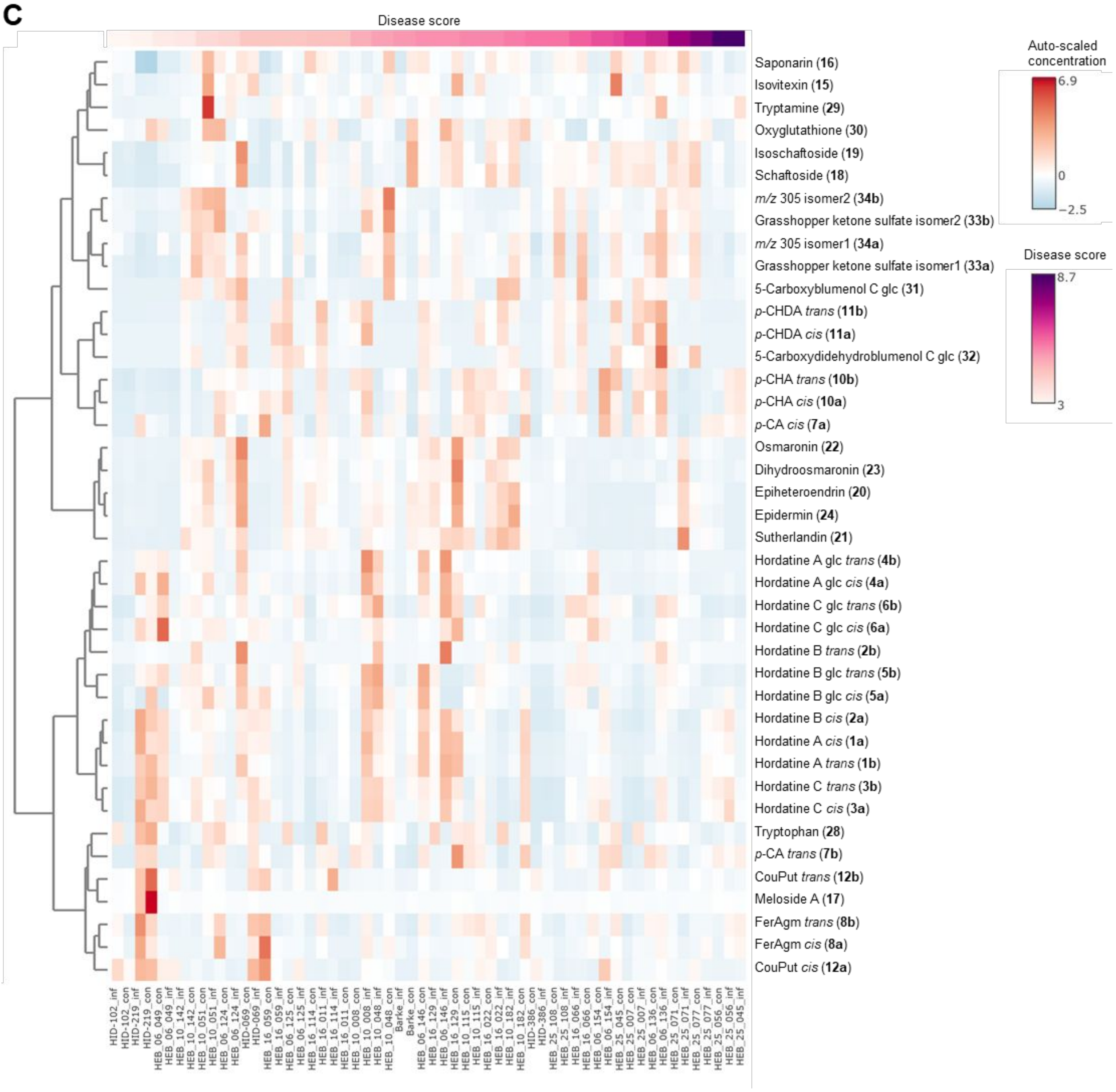

Figure S1. Hierarchical cluster analyses (HCA) of the quantitation of marker metabolites in barley leaves of 29 selected genotypes of the HEB-25 NAM population (1). Sampling of infected (inf) and non-infected control (con) plants was performed (A) 10, (B) 14 and (C) 17 days after inoculation with *B. sorokiniana*.

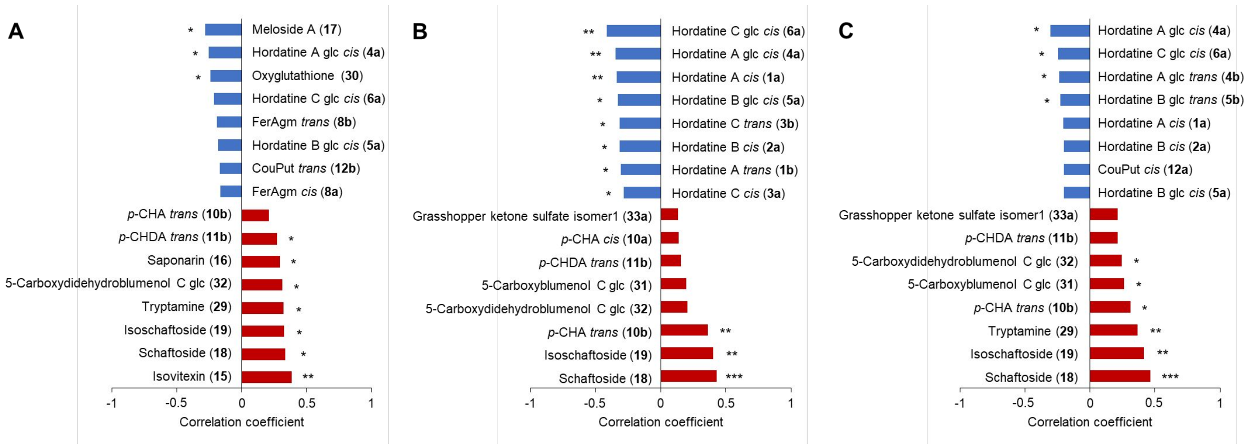

Figure S2. Spearman rank correlation coefficients of the metabolites that most positively (red) and negatively (blue) correlate with the disease score of the *B. sorokiniana* infection. Sampling of infected and non-infected control plants was carried out (A) 10, (B) 14, and (C) 17 days after inoculation with *B. sorokiniana*. *** $p < 0.001$; ** $p < 0.01$; * $p < 0.1$ ($n = 4$).

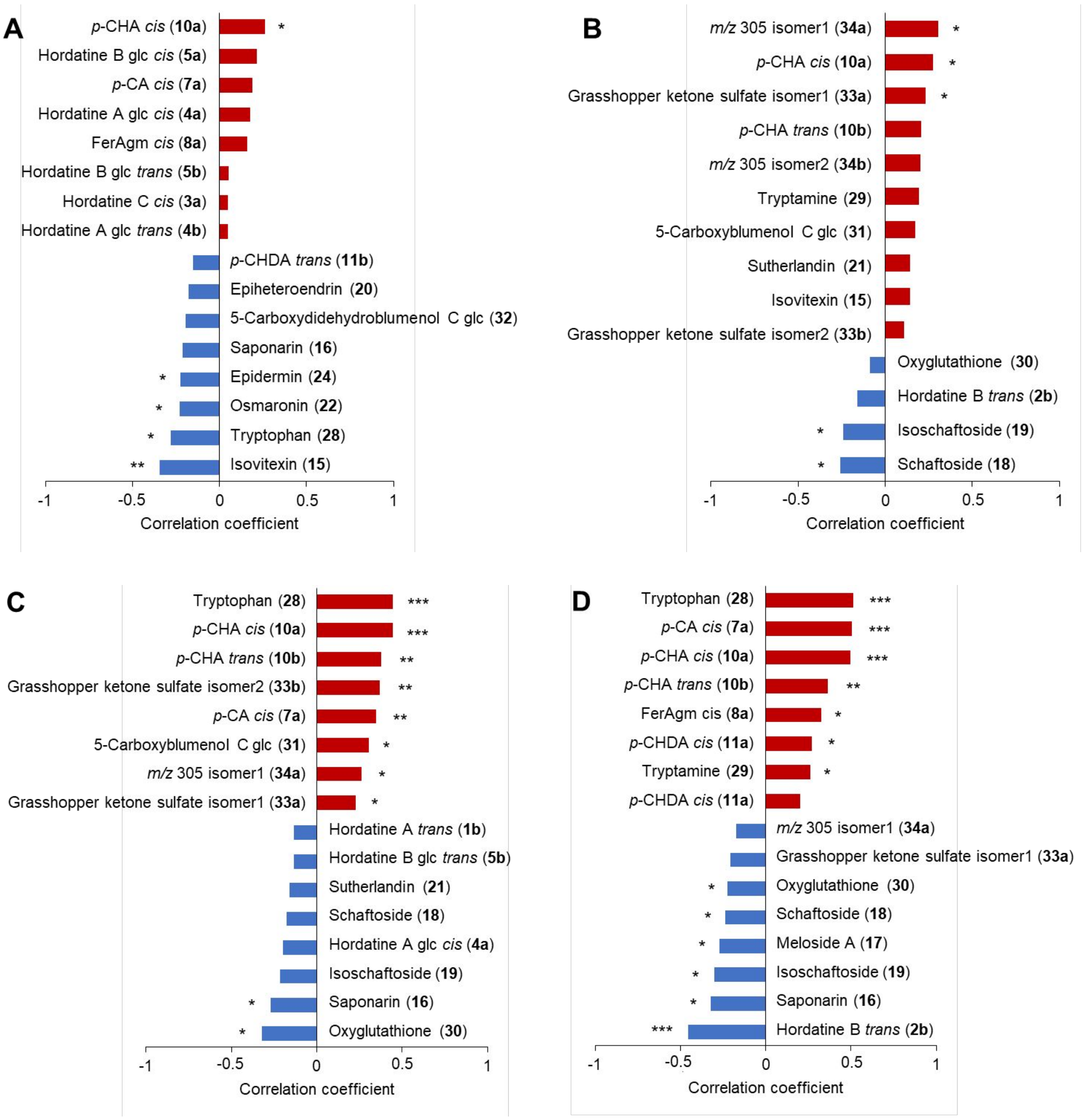

Figure S3. Spearman rank correlation coefficients of the metabolites that are correlated with the infected (red) and non-infected (blue) state. Sampling of infected and non-infected control plants was carried out (A) 7, (B) 10, (C) 14, and (D) 17 days after inoculation with *B. sorokiniana*. *** $p < 0.001$; ** $p < 0.01$; * $p < 0.1$ ($n = 29$).

References

(1) Maurer, A.; Draba, V.; Jiang, Y.; Schnaithmann, F.; Sharma, R.; Schumann, E.; Kilian, B.; Reif, J. C.; Pillen, K. Modelling the genetic architecture of flowering time control in barley through nested association mapping. *BMC Genom.* **2015**, *16*, 290.
